# Diagnosis of Hereditary TTP Caused by Homozygosity for a Rare Complex ADAMTS13 Allele After Salmonella Infection in a 43-Year-Old Asylum Seeker

**DOI:** 10.3389/fmed.2021.639441

**Published:** 2021-02-26

**Authors:** Ralph Wendt, Sven Kalbitz, Felix Otto, Tanja Falter, Joachim Beige, Heidi Rossmann, Bernhard Lämmle

**Affiliations:** ^1^Division of Nephrology and Kuratorium for Dialysis and Transplantation (KfH) Renal Unit, Hospital St. Georg, Leipzig, Germany; ^2^Department of Infectious Diseases, Nephrology and Rheumatology, Hospital St. Georg, Leipzig, Germany; ^3^Institute of Applied Dermatopharmacy, Martin-Luther-University Halle/Wittenberg, Halle, Germany; ^4^Institute of Clinical Chemistry and Laboratory Medicine, University Medical Center Mainz, Mainz, Germany; ^5^Department of Nephrology und Rheumatology, Internal Medicine II, Martin-Luther-University Halle/Wittenberg, Halle, Germany; ^6^Center for Thrombosis and Hemostasis, University Medical Center Mainz, Mainz, Germany; ^7^Department of Hematology and Central Hematology Laboratory, Inselspital Bern University Hospital, University of Bern, Bern, Switzerland; ^8^Haemostasis Research Unit, University College London, London, United Kingdom

**Keywords:** cTTP, Upshaw Schulman syndrome, hereditary TTP, ADAMTS13, TMA

## Abstract

A 43-year-old Armenian patient was diagnosed with salmonella infection and thrombotic microangiopathy (TMA). The clinical course was benign with resolution of all laboratory alterations after antibiotic treatment. Constantly deficient ADAMTS13 activity without ADAMTS13 inhibitors and evidence of homozygosity for a rare complex ADAMTS13 allele led to the diagnosis of congenital thrombotic thrombocytopenic purpura (cTTP). Half-life of ADAMTS13 after plasma infusion was calculated (27,6h) and double blinded plasma infusion as well as ergometric exercise with and without prior plasma infusion undertaken to investigate suspected smoldering TTP activity.

## Introduction

Congenital or hereditary thrombotic thrombocytopenic purpura (cTTP) or Upshaw Schulman syndrome (USS, OMIM #274150) is a very rare disorder characterized by severe ADAMTS13 deficiency. In the vast majority TTP is caused by autoantibodies toward ADAMTS13 (a disintegrin and metalloprotease with thrombospondin type 1 repeats, member 13) with consecutive ADAMTS13 deficiency. In cTTP, ADAMTS13 deficiency is due to mutations in the *ADAMTS13* gene. It is inherited as an autosomal recessive trait and accounts for <10% of all TTP cases. ADAMTS13 protease deficiency leads to persistence of ultra large von Willebrand factor multimers and eventually to acute thrombotic microangiopathy (TMA). Most cTTP cases are diagnosed in childhood or early adolescence after a first acute TTP episode. Here, we present an unusual case of late-onset cTTP occurring after salmonella infection at the age of 43 years caused by homozygosity for a rare, complex ADAMTS13 allele.

## Case Presentation

**Figure 1:**
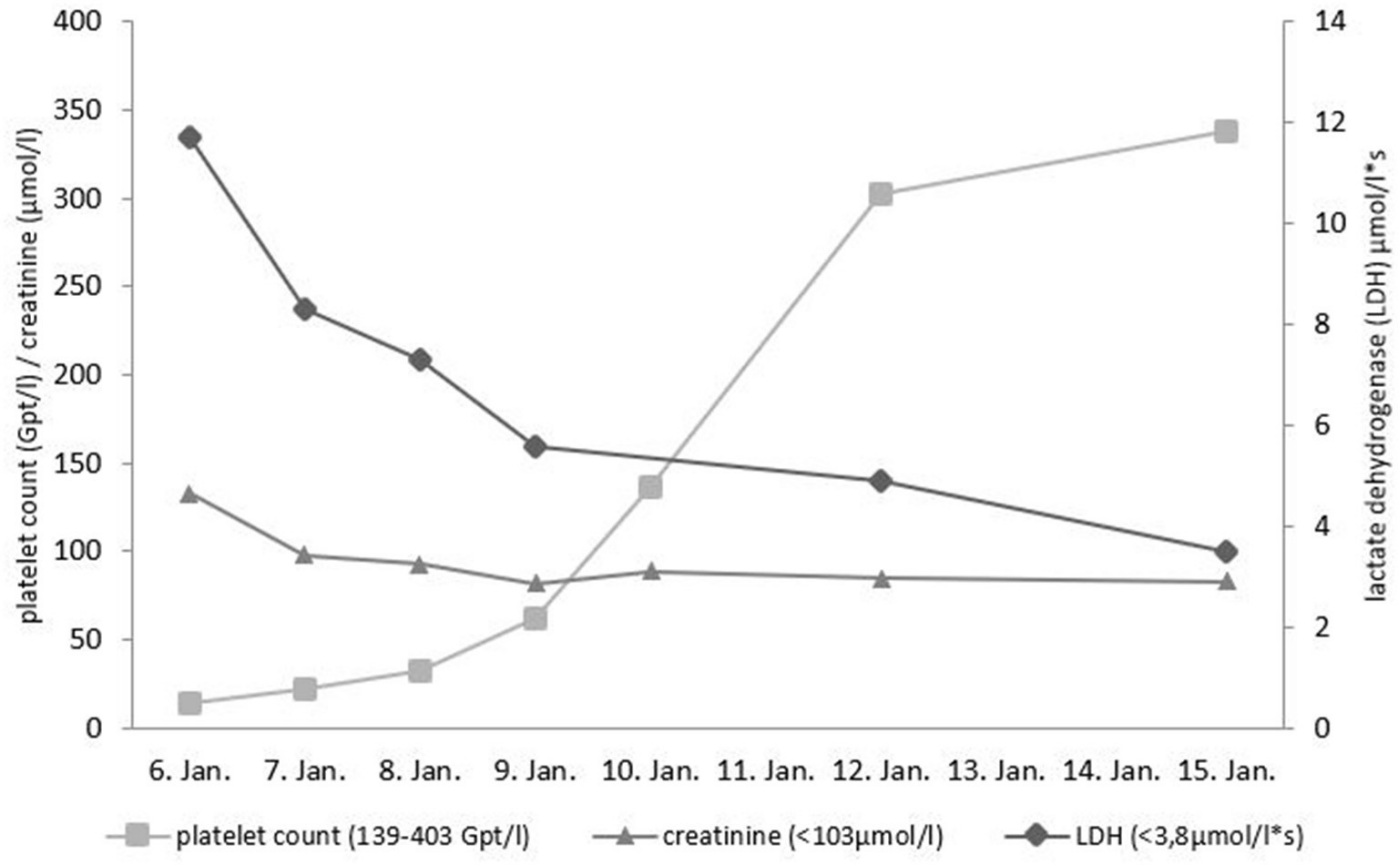
Course of platelet count, lactate dehydrogenase (LDH) and creatinine during initial presentation. Reference ranges given in parentheses.

A 43-year-old person of Armenian ancestry presented to our hospital due to fever and severe diarrhea. This patient was an asylum-seeking refugee in Germany, who just recently had a barbecue. Salmonella enteritidis enterocolitis was diagnosed with associated acute kidney injury AKIN I. There was severe thrombocytopenia (14 GPT/l) and signs of hemolysis (elevated lactate dehydrogenase level, low haptoglobin) (Figure 1). The initial differential diagnosis of the treating colleagues at the Infectiology department was Shiga toxin-producing E. coli induced hemolytic-uremic syndrome (STEC-HUS) due to the presentation with diarrhea. Based on the result of a stool culture revealing Salmonella enterica the attending physician suspected a Salmonella-induced secondary HUS. The patient received supportive treatment (volume replacement with balanced electrolyte solutions) and antibiotic treatment for salmonella (azithromycin). Platelet count normalized within 6 days, renal function completely normalized, and all other laboratory abnormalities were also resolved (Figure 1). Due to diagnosis of latent tuberculosis (based on positive QuantiFERON-test without evidence of active tuberculosis) the patient was treated with isoniazid for nine months.

The patient received neither plasma products nor plasma exchange at any time point during hospitalization.

The diagnosis of TTP was made after consulting with the nephrologist and after normalization of laboratory findings and resolution of the clinical symptoms. There was no measurable ADAMTS13 activity or ADAMTS13 antigen during the acute phase of the disease and no evidence of anti-ADAMTS13 IgG antibodies. ADAMTS13 deficiency was shown to be persistent during the follow-up (Table 1). Because of suspected cTTP, a genetic analysis of *ADAMTS13* gene was performed.

**Table 1:** ADAMTS13 activity and inhibitor at presentation and during follow-up.

	**09.01.2018**	**15.03.2018**	**15.10.2018**	**01.11.2018**
ADAMTS13 activity, % (40–130)	<3.0^*^	<3.0^*^	<1.0^**^	<1.0^**^
ADAMTS13 concentration, μg/ml (0.31−0.82)	<0.01^*^			
ADAMTS13 inhibitor, Bethesda Units (<0.5)			0^**^	0^**^
ADAMTS13 IgG antibodies, U/ml (<12)	1.1^*^			

* Values measured at Institute of Transfusion Medicine and Clinical Hemostasiology, St. Georg Hospital Leipzig, Germany (Technozym^®^ ADAMTS13 ELISA Kit and TECHNOZYM ADAMTS13 INH, measuring anti-ADAMTS13 antibodies by ELISA)

** *ADAMTS13 activity and functional inhibitor measured by in house assay using the FRETS-VWF73 substrate (1, 2) at the Institute of Clinical Chemistry and Laboratory Medicine, University Medical Center Mainz, Germany*.

## Results

### Family History

The parents of our patient had died at the age of 78 years (mother, unknown cause) and 75 years (father, “stroke”). The patient has 2 brothers (52 and 56 years old) and five sisters (46, 51, 55, 57, and 62 years of age), who are refugees (currently in Russia) and unfortunately not accessible for us despite all efforts. Another brother had died at the age of 22 years from suspected stroke. Two sisters are known to have heart problems (no objective data available). According to our patient there was no other sibling with any severe disorder.

### Laboratory Values During the Disease Course and Diagnosis of cTTP

The patient has been subjected to a series of tests in order to clarify his symptoms and to optimize his clinical management.

The course of the most significant laboratory analyses are depicted in Table 1. No plasma products were given and no plasmapheresis was performed. The only treatment was oral antibiotic therapy of salmonella infection and intravenous volume replacement.

Repeated analyses of ADAMTS13 activity and inhibitor or antibodies, respectively, showed no measurable protease activity and no evidence for ADAMTS13 antibodies at any time.

### Genetic Analysis

Sanger sequencing of the *ADAMTS13* (OMIM: ^*^604134; RefSeq: NM_139025.4) exons and splice sites was performed on a CEQ 8000 Genetic Analysis System (Sciex, Darmstadt, Germany). In exon 19 the variant c.2351G>A, p.Arg784Gln (dbSNP rs377187626), in exon 22 the variant c.2746C>T, p.Arg916Cys (dbSNP rs374444423) were detected, both in homozygosity. A large deletion of the *ADAMTS13* gene was excluded by NGS (Next Generation Sequencing): Target enrichment was achieved by in solution custom probe hybridization (IDT, Leuven, Belgium). Sequencing was performed by a MiSeq instrument (Illumina, Berlin, Germany) confirming homozygosity for the complex allele c.[2351G>A;2746C>T] p.[Arg784Gln;Arg916Cys].

The patient's son was found to be heterozygous for c.[2351G>A;2746C>T] p.[Arg784Gln;Arg916Cys] and had an ADAMTS13 activity of 48.6%.

### Plasma Infusion Trial

**Figure 2:**
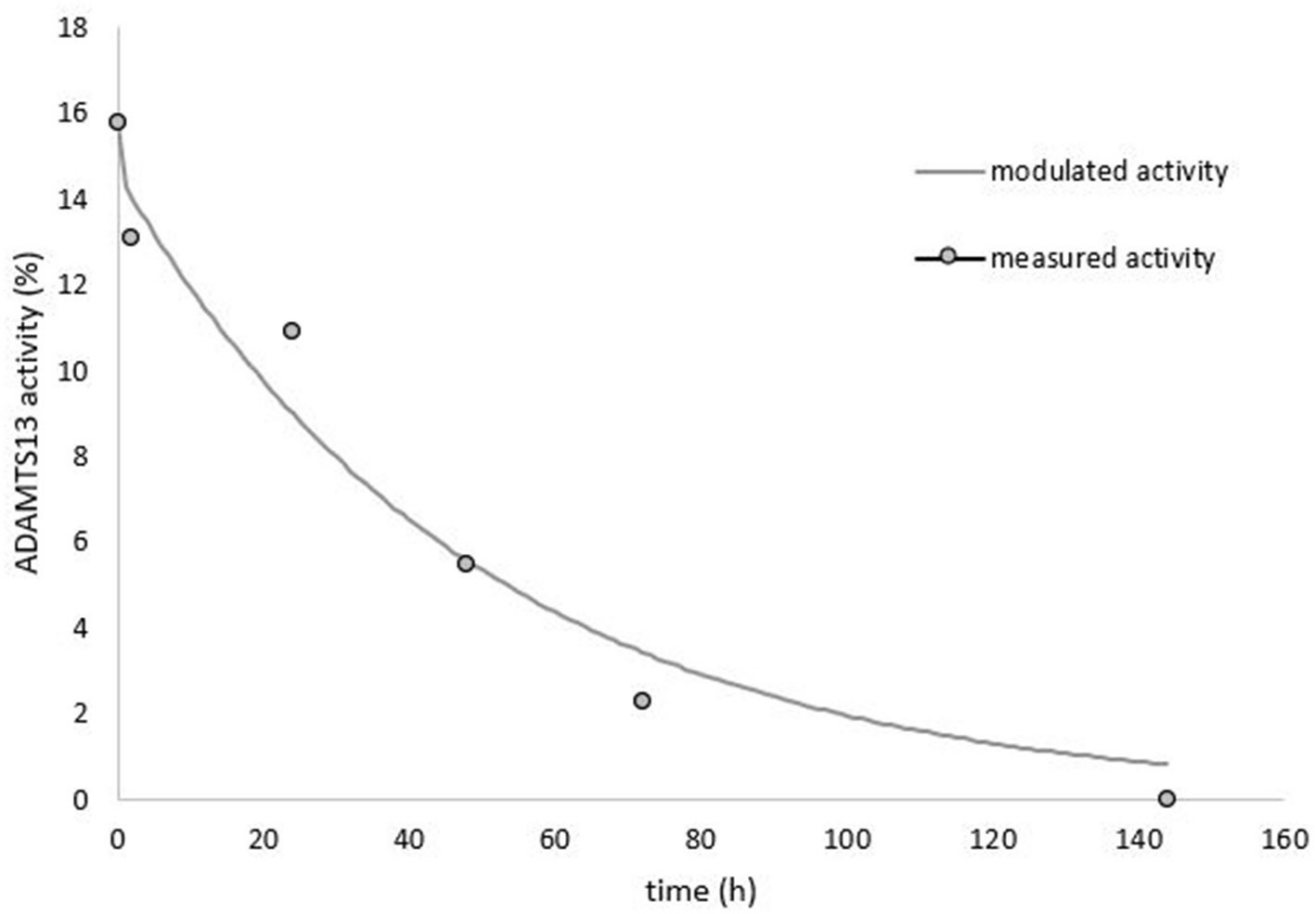
ADAMTS13 activity measured over time after 9 ml/kg plasma infusion.

To further confirm cTTP we calculated plasma half-life of ADAMTS13 after donor plasma infusion (Figure 2). The single time infusion volume was 9 ml/kg bodyweight (BW) fresh frozen plasma (FFP) with a pooled ADAMTS13 activity of 85%. T_0_ refers to the time at which the plasma volume was fully infused (2 h after start of the infusion). ADAMTS13 activity at T_0_ was 15.7%. Based on the predicted plasma volume of 3.72 liters, we calculated a maximum ADAMTS13 activity of 18.6%. This value corresponds well to the extrapolated protease activity of 17.1% at 1 h after plasma infusion. Additional blood samples for ADAMTS13 activity were drawn at 2, 24, 48, 72, and 144 h after end of the infusion. A fast initial decline in ADAMTS13 activity was observed between 0 and 2 h, which afterwards changed into a first order elimination kinetics. Therefore, we calculated the ADAMTS13 activity half-life as 27.6 h (95% confidence interval: 19.9–45.12 h) according to a 2-phase elimination kinetics (3).

### Investigation of Smoldering Clinical TTP Manifestations

#### Maximal Bicycle Exercise Testing With and Without Prior Plasma Infusion

**Figure 3:**
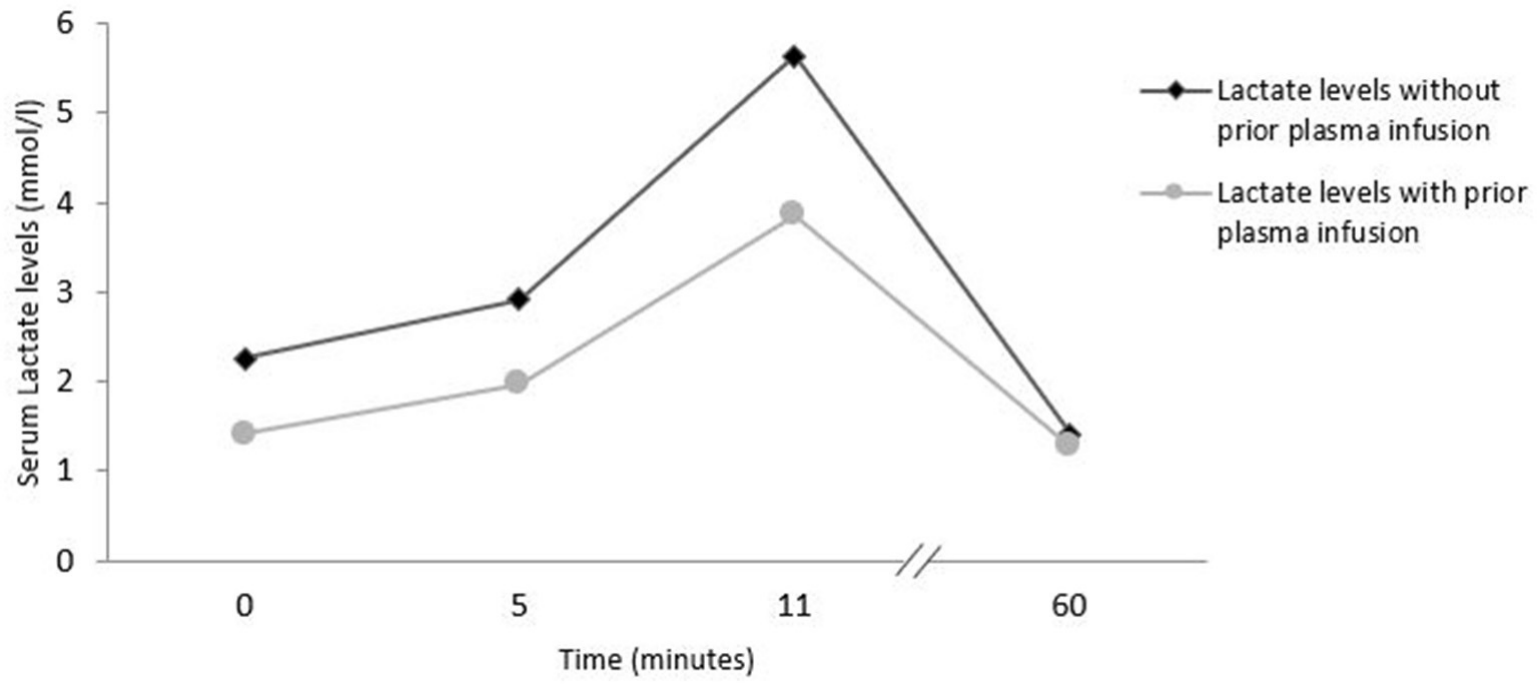
Exercise induced lactate levels in bicycle exercise testing with and without prior plasma infusion.

Usual bicycle exercise testing (ergometry) was performed with blood samples taken at the following timepoints: start of ergometry, 5 min after start, at the end of exercise and 60 min after start of exercise (Figure 3). The first ergometry testing was performed without prior plasma infusion. Exercise was started at 50 watts and increased every 2 min by 25 watts. After almost 11 min and at 150 watts, the ergometric exercise was stopped due to patient exhaustion. 48 h later a second ergometry was performed after prior infusion of 525 ml fresh frozen plasma. Laboratory assays at the different time points of exercise testing are shown in Supplementary Table 1.

#### Double Blinded Infusion Trial (Crystalloids Vs. Fresh Frozen Plasma)

On the day of our blinded infusion trial, the patient repeatedly reported thoracic (6/10), abdominal (5/10) und back pain (8/10), quantified on a 10-point visual analog pain scale (VAPS). We blinded the patient, the treating physicians and the nurses by using aluminum foils for the infusion lines and infusion fluids (Supplementary Figure 1). Infusion time was set to 2 h. We started with two parallel infusions of 500 ml each of balanced electrolyte fluids (E153), followed by two parallel infusions of 250 ml each of fresh frozen plasma.

After the infusion of the balanced electrolyte solution, thoracic pain (starting from 6/10 on the VAPS) and abdominal pain (starting from 5/10 on the VAPS) completely vanished (both 1/10 VAPS), but back pain persisted at 8/10 on the VAPS (Supplementary Table 2). After plasma infusion the back pain was almost completely gone (2/10 VAPS).

## Discussion

Congenital TTP (or Upshaw-Schulman syndrome) is a very rare, autosomal recessive disease, which is caused by homozygous or compound heterozygous *ADAMTS13* mutations (4–14). The diagnosis is confirmed by evidence of a persistent severe ADAMTS13 deficiency (<10%), absence of an ADAMTS13 inhibitor and presence of biallelic mutations of *ADAMTS13*. There seems to be no clear genotype-phenotype relationship (4). Subjects, e.g., parents, heterozygous for an *ADAMTS13* mutation are unaffected, also under conditions that can precipitate TTP episodes. The son of our patient was heterozygous for the patient's mutation and showed ADAMTS13 activity of about 50%, which seems sufficient to prevent acute TTP episodes.

The patient is homozygous for the complex allele c.[2351G>A;2746C>T] p.[Arg784Gln;Arg916Cys]. Both variants, Arg784Gln in Exon 19 (dbSNP: rs377187626) and Arg916Cys in Exon 22 (dbSNP: rs374444423) are very rare (minor allele frequency of both <0.00008 [dbSNP]).

Though analysis by bioinformatic tools is inconsistent (Mutation Taster: polymorphism; PolyPhen2: probably damaging; SIFT: tolerated; PROVEAN: deleterious), a biological consequence of c.2746C>T, p.Arg916Cys is plausible as it creates an unpaired cysteine. More importantly, it was reported in compound heterozygosity with a further *ADAMTS13* mutation in several Upshaw-Schulman syndrome (USS) patients (15–17). Furthermore, a poster presented by Russo et al. (18), reported an USS patient with homozygosity for the same complex allele as found in the patient described here.

In summary, we consider the complex allele Arg784Gln; Arg916Cys to be the cause of our patient's disease, but it remains unclear whether Arg784Gln, which is classified as benign by the applied bioinformatic tools (Mutation Taster, PolyPhen2, SIFT, PROVEAN), contributes to the allele's pathogenicity.

Since no residual plasma ADAMTS13 activity was detected, we conclude that Arg784Gln;Arg916Cys causes a severe defect of the affected *ADAMTS13* allele, suggesting that either no protein is expressed, protein maturation is severely impaired, or the protein is not secreted.

### Plasma Half-Life of ADAMTS13

In patients with autoimmune TTP (iTTP) there is usually no measurable increase in ADAMTS13 activity after plasma infusion. Therefore, the increase of ADAMTS13 activity in our patient after plasma infusion confirms our diagnosis of cTTP. The ADAMTS13 activity half-life of 27.6 h in our patient is somewhat shorter than most previous reported elimination half-life values.

The expected maximal concentration of ADAMTS13 activity at the beginning of the infusion [calculated via equation described by Furlan et al. (19)] was 18.6%. The fast decline of ADAMTS13 activity at the beginning of the curve points to a multiple-compartment kinetics. Few studies have documented the same effect after plasmatherapy, but this can mostly be attributed to the lack of data points early after infusion. The half-life (t_1/2_) of ADAMTS13 reported in the scarce literature is significantly longer [between 2.1 days [50.4 h] up to 7,9 days [189.5 h] (19–21)].

The plasma half-life of ADAMTS13 in another case of cTTP was close to our findings with an elimination half-life of 1.54 days (37 h) (22). We suppose that part of the infused ADAMTS13 will be trapped by binding to endothelial cell-attached VWF multimers. There seems to be no significant difference between ADAMTS13 half-life of cTTP patients with prior plasma exchange vs. patients just receiving plasma infusions (21).

The shorter ADAMTS13 half-life in our case may also be related to the multi-compartment elimination, as previous treatments with plasma infusions or plasmapheresis over an extended time period could saturate all compartments of ADAMTS13 and cause higher ADAMTS13 half-life measurements.

In the study of the pharmacokinetics of recombinant ADAMTS13 (rhADAMTS13) the t_1/2_ was 2,47 d (59.2 h) with a reported initial half-life of 17 h (23). In one patient the half-life was as low as 29.5 h, comparable to the result in our patient.

There is a rather large variation of reported half-lives due to a paucity of systematic testing.

### Late Onset cTTP

Several factors (most of them not understood) influence the severity of disease and first occurrence of an acute TTP episode. An important factor is residual ADAMTS13 activity (24). But results of the large International Hereditary TTP Registry with 123 enrolled cTTP patients showed that residual ADAMTS13 activity is not the only determinant of the age at first disease manifestation (4). Median age at overt disease onset was 4.52 years and at clinical diagnosis 16.7 years. Another important factor is exposition to strong triggers of disease (infections, surgery, pregnancy, drugs, etc.).

The influence of AB0 blood group type on plasma levels of von Willebrand factor (VWF) is well-documented in several studies (25–27). A large study with healthy individuals nicely showed that plasma VWF levels were lowest in blood group 0 persons and highest in blood group AB subjects (28). Among patients from the Oklahoma TTP registry with acquired TTP blood group 0 was more frequent than expected and was even discussed to be a potential risk factor (29). While blood group might play a role in iTTP, there are no data in USS patients. Whether blood group 0 may protect cTTP patients from early disease onset is unknown.

The first disease onset and clinical diagnosis in our patient was at the age of 43 years. Our patient was carrier of two homozygous *ADAMTS13* variants and had no residual plasma ADAMTS13 activity, his blood group was 0.

There was no anamnestic finding that could retrospectively be attributed to an earlier TTP episode. Because of the complete resolution of the TTP flare by just treating the triggering situation in this case, we cannot exclude earlier unknown disease manifestations which may have resolved by treating the underlying triggering condition without diagnosis of TTP.

### Smoldering TTP Activity

In the literature there are hints to ongoing disease activity despite lack of classical signs and symptoms of active disease (4, 5, 30, 31). Symptoms reported were headaches, loss of concentration or abdominal discomfort (5), which were reversed by prophylactic plasma infusions.

Our patient consistently reported thoracic and abdominal pain. There were no pathological changes in ECG, transthoracic echocardiography, abdominal sonography or repeated troponin values, lipase, lactate dehydrogenase and other parameters. There were no laboratory signs of TTP flare. The most interesting observation was a complete resolution of all pain burden within 2 h of plasma infusion. The positive effect lasted for about 4 days, as reported by the patient without any knowledge nor information about half-life of ADAMTS13 or advanced knowledge of the disease. We could reproduce this effect of complete pain resolution on two further occasions. With this information we searched for signs of “smoldering TTP activity,” but we could not find any laboratory value which was consistently or temporarily altered (troponin, lipase, LDH, haptoglobin, CK, platelets, lactate, coagulation parameters including D-dimers). We tried to provoke signs of smoldering clinical TTP with clinical provocation tests that could possibly cause peripheral ischemia. We did maximum ergometric cycling exercise with and without prior prophylactic plasma infusion, but–except for a lower lactate at starting point–we were unable to find any difference in any parameter evaluated: no significant differences in dynamics of troponin, lipase, LDH, haptoglobin, CK, platelets and lactate values (Figure 3, Supplementary Table 1) between the two ergometry tests.

In the further course the patient reported spontaneous disappearance of the above-named symptoms without any intervention. When the symptoms returned, we decided to do the double-blinded infusion trial with balanced crystalloid fluids vs. fresh frozen plasma infusions with an impressive effect of just crystalloid fluids on thoracic and abdominal pain. The fresh frozen plasma was then effective in reducing back pain. It is difficult to interpret these findings correctly and we rather doubt having proof of smoldering TTP activity reacting exclusively to replenishment of ADAMTS13 via plasma infusion (5, 6, 31). There seems to be a significant placebo effect, especially in this patient with a huge language barrier and fear of deportation to his home country after possible failure of asylum procedures and expiration of residency permit.

### Triggers of Acute Episodes of cTTP

The salmonella infection was clearly the trigger for the acute TTP flare in our case.

It is nevertheless remarkable that the acute TTP episode was completely resolved just by treating the trigger situation (antibiotics against salmonella, supportive treatment) without any plasma infusion. Platelet count normalized on day 6 and lactate dehydrogenase on day 9 after presenting at our hospital (day 4 resp. day 7 after starting antibiotic therapy against salmonellosis). The inflammatory trigger (salmonella infection) probably caused an upregulation of endothelial VWF secretion and increase of VWF plasma levels, which caused acute flare of the disease in analogy to triggering acute TTP by shiga toxin in an ADAMTS13 deficient mouse model (32).

Until recombinant ADAMTS13 (23) becomes available, treatment is either on demand with plasma infusions during acute flares of TTP (10 to 20 ml/kg BW of fresh frozen plasma) or prophylactic with 10–15 ml/kg BW every 2–3 weeks. Data from the International hereditary TTP registry show that 71% of the 117 registered cTTP patients with available treatment data receive prophylactic plasma infusions and just 29% on demand treatment (4). We decided for “on demand” treatment strategy and careful surveillance in our patient. In the 3 years of follow-up since diagnosis, there has been no recurrence of TTP episodes. Echocardiography, myocardial scintigraphy, and cerebral MRI were performed without pathological findings.

Although our understanding of cTTP has increased enormously over the last two decades, there is still a lack of knowledge about long-term sequelae and possible silent smoldering end organ damage, which makes lifelong careful follow-up and regular assessments imperative.

## Data Availability Statement

The original contributions presented in the study are included in the article/Supplementary Material, further inquiries can be directed to the corresponding author/s.

## Ethics Statement

Ethical review and approval was not required for the study on human participants in accordance with the local legislation and institutional requirements. The patients/participants provided their written informed consent to participate in this study. Written informed consent was obtained from the individual(s) for the publication of any potentially identifiable images or data included in this article.

## Author Contributions

RW: diagnosing the patient, conducting clinical tests, writing, and revision of the manuscript. BL: senior advisor in all laboratory, genetical and clinical analyses, and manuscript revision. HR: laboratory analyses of ADAMTS13 and inhibitors, genetic analysis and interpretation, and manuscript revisions. JB: clinical testing and follow up and manuscript revision. TF: manuscript revision. FO: pharmacokinetic analyses and revision of manuscript. SK: caring for the patient and revision of manuscript. All authors contributed to the article and approved the submitted version.

## Conflict of Interest

The authors declare that the research was conducted in the absence of any commercial or financial relationships that could be construed as a potential conflict of interest.

## Supplementary Material

Supplementary Figure 1 Blinded assessment of symptoms using aluminum foil-covered infusion fluids and lines.

Supplementary Table 1 Laboratory values during bicycle exercise testing with and without prior plasma infusion. Values of ergometry without prior plasma infusion are in black, values of ergometry with prior plasma infusion are in gray. Lactate (<2.4 mmol/l), D-dimer (<500 μg/l), troponin (<0.005 μg/l), platelets count (139 – 403 Gpt/l), lipase (<1.0 μkat/l), lactate dehydrogenase (2.2–3.8 μmol/l^*^s), creatine kinase (CK) (<3.2 μmol/l^*^s), haptoglobin (hapto) (0.2–2.04 g/l). D-dimers in the “with prior plasma-infusion” setting were elevated most likely due to clinically apparent thrombophlebitis.

Supplementary Table 2 10-point visual analog pain scale (VAPS) at baseline and after infusion of crystalloids and FFP.
