# Supplementary figures and images for: Diagnosis of Hereditary TTP Caused by Homozygosity for a Rare Complex ADAMTS13 Allele After Salmonella Infection in a 43-Year-Old Asylum Seeker
Source: Front Med (Lausanne). 2021 Feb 26;8:639441. doi: 10.3389/fmed.2021.639441 (PMC7959797; doi:10.3389/fmed.2021.639441)

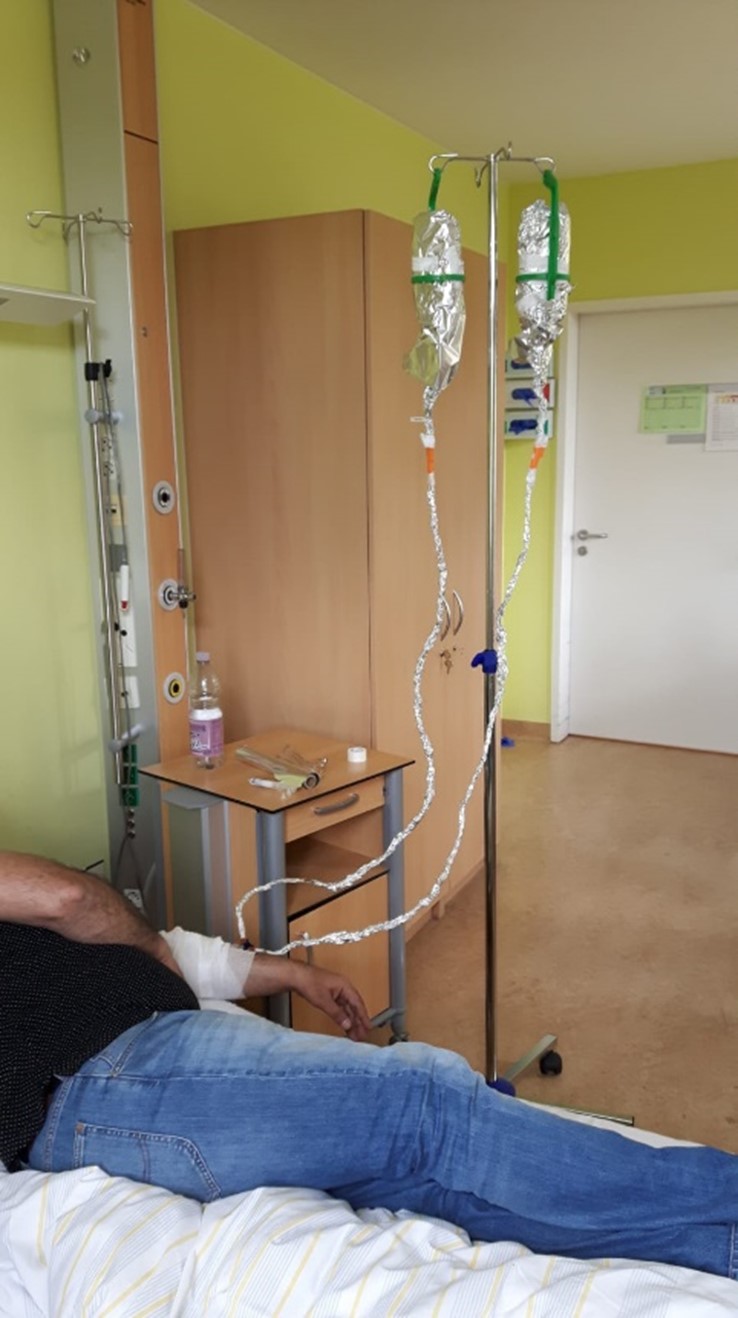

Supplement: Supplementary Figure 1 — Blinded assessment of symptoms using aluminum foil-covered infusion fluids and lines. [file Image_1.jpg]
