# Supplementary material for: Diagnosis of Hereditary TTP Caused by Homozygosity for a Rare Complex ADAMTS13 Allele After Salmonella Infection in a 43-Year-Old Asylum Seeker
Source: Front Med (Lausanne). 2021 Feb 26;8:639441. doi: 10.3389/fmed.2021.639441 (PMC7959797; doi:10.3389/fmed.2021.639441)
Supplement: Supplementary Table 1 — Laboratory values during bicycle exercise testing with and without prior plasma infusion. Values of ergometry without prior plasma infusion are in black, values of ergometry with prior plasma infusion are in gray. Lactate (<2.4 mmol/l), D-dimer (<500 μg/l), troponin (<0.005 μg/l), platelets count (139 – 403 Gpt/l), lipase (<1.0 μkat/l), lactate dehydrogenase (2.2–3.8 μmol/l*s), creatine kinase (CK) (<3.2 μmol/l*s), haptoglobin (hapto) (0.2–2.04 g/l). D-dimers in the “with prior plasma-infusion” setting were elevated most likely due to clinically apparent thrombophlebitis. [file Table_1.docx]

|  | lactate | lactate | D-dimer | D-dimer* | troponin | troponin | platelets | platelets | lipase | lipase | LDH | LDH | CK | CK | hapto | hapto |
| --- | --- | --- | --- | --- | --- | --- | --- | --- | --- | --- | --- | --- | --- | --- | --- | --- |
| start (0min) | 2,26 | 1,42 | 169 | 799 | <0,005 | <0,005 | 248 | 294 | 0,5 | 0,7 | 3 | 3 | 3,3 | 2,4 | 0,8 | 0,8 |
| after 5min | 2,92 | 1,97 | 243 | 809 | <0,005 | <0,005 | 254 | 272 | 0,5 | 0,6 | 3 | 3 | 3,3 | 2,4 | 0,8 | 0,8 |
| end (11min) | 5,64 | 3,86 | 414 | 1483 | <0,005 | <0,005 | 226 | 298 | 0,5 | 0,6 | 3 | 4 | 3,3 | 2,5 | 0,8 | 0,9 |
| 60min | 1,42 | 1,28 | 169 | 1851 | <0,005 | <0,005 | 267 | 292 | 0,5 | 0,6 | 3 | 3 | 3,3 | 2,7 | 0,8 | 0,9 |
