# Supplementary material for: Diagnosis of Hereditary TTP Caused by Homozygosity for a Rare Complex ADAMTS13 Allele After Salmonella Infection in a 43-Year-Old Asylum Seeker
Source: Front Med (Lausanne). 2021 Feb 26;8:639441. doi: 10.3389/fmed.2021.639441 (PMC7959797; doi:10.3389/fmed.2021.639441)
Supplement: Supplementary Table 2 — 10-point visual analog pain scale (VAPS) at baseline and after infusion of crystalloids and FFP. [file Table_2.docx]

|  | baseline | after crystalloids | after FFP |
| --- | --- | --- | --- |
| Thoracic pain | 6/10 | 1/10 | 1/10 |
| Abdominal pain | 5/10 | 1/10 | 1/10 |
| Back pain | 8/10 | 8/10 | 2/10 |
